# Accumulation of Polychlorinated Biphenyls in Adipocytes: Selective Targeting to Lipid Droplets and Role of Caveolin-1

**DOI:** 10.1371/journal.pone.0031834

**Published:** 2012-02-20

**Authors:** Sophie Bourez, Soazig Le Lay, Carine Van den Daelen, Caroline Louis, Yvan Larondelle, Jean-Pierre Thomé, Yves-Jacques Schneider, Isabelle Dugail, Cathy Debier

**Affiliations:** 1 Institut des Sciences de la Vie, UCLouvain, Louvain-la-Neuve, Belgium; 2 Université Pierre et Marie Curie – Paris 6, UMR S 872, Paris, France; 3 Laboratoire d'Ecologie animale et d'Ecotoxicologie, Université de Liège, Liège, Belgium; National Institutes of Health, United States of America

## Abstract

**Background:**

Polychlorinated biphenyls (PCBs) are persistent environmental pollutants that preferentially accumulate in lipid-rich tissues of contaminated organisms. Although the adipose tissue constitutes a major intern reservoir of PCBs and recent epidemiological studies associate PCBs to the development of obesity and its related disorders, little is known about the mechanisms involved in their uptake by the adipose tissue and their intracellular localization in fat cells.

**Methodology/Principal Findings:**

We have examined the intracellular distribution of PCBs in mouse cultured adipocytes and tested the potential involvement of caveolin-1, an abundant adipocyte membrane protein, in the uptake of these compounds by fat cells. We show that 2,4,4′-trichlorobiphenyl (PCB-28), 2,3′,4,4′,5-pentachlorobiphenyl (PCB-118) and 2,2′,4,4′,5,5′-hexachlorobiphenyl (PCB-153) congeners rapidly and extensively accumulate in 3T3-L1 or mouse embryonic fibroblast (MEF) derived cultured adipocytes. The dynamics of accumulation differed between the 3 congeners tested. By subcellular fractionation of primary adipocytes, we demonstrate that these pollutants were almost exclusively recovered within the lipid droplet fraction and practically not associated to cell membranes. The absence of caveolin-1 expression in primary adipocytes from cav-1 deficient mice did not modify lipid droplet selective targeting of PCBs. In cav-1 KO MEF differentiated adipocytes, PCB accumulation was decreased, which correlated with reduced cell triglyceride content. Conversely, adenoviral mediated cav-1 overexpressing in 3T3-L1 cells, which had no impact on total cell lipid content, did not change PCB accumulation.

**Conclusion/Significance:**

Our data indicate that caveolin-1 *per se* is not required for selective PCB accumulation, but rather point out a primary dependence on adipocyte triglyceride content. If the crucial role of lipid droplets in energy homeostasis is considered, the almost exclusive accumulation of PCBs in these organelles warrants future attention as the impairment of their function could be linked to the worldwide obesity epidemic.

## Introduction

Polychlorinated biphenyls (PCBs) are persistent environmental pollutants that are found at elevated concentrations in the adipose tissue of contaminated organisms [1]. These organic contaminants have shown numerous harmful effects on animal and human health [2], [3]. Moreover, there is increasing information in the literature correlating the presence of PCBs in contaminated organisms and the development of obesity related disorders such as type II diabetes and cardiovascular diseases [4]–[6], and suggesting a putative role for these chemicals in the fundamental mechanisms controlling the adipose tissue metabolism and obesity pandemy. A recent study demonstrated increased persistent organic pollutant (POP) total body burden in obese subjects and increased POP serum concentrations after bariatric surgery leading to drastic weight loss [7]. Conversely, how these compounds interfere with metabolic regulation remains poorly understood [4], [7], [8].

It is however clearly established that adipose tissues are preferential sites for PCB accumulation [1], [9]. Due to their lipophilic properties, at least two preferential cellular compartments are likely candidates for their accumulation: polar lipids of biological membranes or the lipid droplet, which contains the most important part of adipocyte triglyceride stores.

Although very little is known about mechanisms that lead to the preferential targeting of PCBs towards adipose tissues, some recent experimental data might suggest a role for caveolae in this process. Caveolae are 50–100 nm invaginations in the plasma membrane that are particularly abundant in endothelial cells, muscle cells and adipocytes [10]. The caveolar membrane is invaginated by a major coat constituent, caveolin, and its composition is closely related to that of lipid rafts, enriched in cholesterol and sphingolipids. The caveolin family comprises three related members of intramembrane proteins, caveolin-1, -2 and -3, which mostly differ in their tissue distribution. Caveolin-1 and -2 are coexpressed and are especially abundant in the adipose tissue and vascular endothelium, whereas caveolin-3 is muscle restricted [11].

Several lines of evidence indicate that caveolae/caveolins might be critical adipose cell components leading to an intracellular accumulation of PCBs. Firstly, caveolae formation was shown to be induced by some PCB congeners, suggesting that caveolae could be involved in the uptake of toxic, lipophilic xenobiotics by endothelial cells [12], [13]. Secondly, specific receptors for albumin and certain lipoproteins (high-density lipoproteins and oxidized low-density lipoproteins), which participate to PCB transport in plasma [14], [15], are localized in caveolae [16]. Thirdly, endothelial cell exposure to 3,3′,4,4′-tetrachlorobiphenyl (PCB-77), induced specific accumulation of this congener in the caveolae-rich fraction of cellular membranes [13]. Fourthly, fat cells, in which PCBs preferentially accumulate *in vivo*, contain numerous caveolae [10], [17]. Fifthly, caveolin-1 is dually distributed between the cell surface and the lipid droplet organelle in adipocytes, and exogenous lipid addition to cultured adipocytes directs caveolin-1 to the lipid droplet pool, suggesting a role for lipophilic molecules in the stimulation of caveolar endocytosis [18]–[20]. Thus, caveolin trafficking between the plasma membrane and lipid droplet pools [20] might regulate PCB intracellular distribution.

In the present study, we investigated the intracellular localization of 3 PCB-congeners (PCB-28, -118, -153) in the adipose tissue and the possible involvement of the caveolae and caveolin-1 in their entry in fat cells. We used diverse adipocyte cell culture systems to evaluate the dynamics of PCB uptake by adipocytes *in vitro*. Our results show a massive accumulation of these pollutants in fat cells and their almost exclusive association with the lipid droplet compartment. The dynamics of entry however differed between the 3 congeners investigated. In a second step, we modulated the expression of caveolin-1 to examine the role of this protein in both the uptake and the targeting of PCBs to lipid droplets. Our data identify the adipocyte neutral lipids as major determinants of PCB accumulation in fat cells, a process that does not require caveolin-1.

## Materials and Methods

### Animal tissues

Caveolin-1 KO mice and their wild type littermates, maintained in the animal facility, were described previously [20]. All animal experiments were approved by local authorities in accordance with the criteria outlined by the French veterinary guidelines. The study was approved by the Regional Ethics Committee for Animal experiment, N°3 of Ile de France, Paris; Approval ID : p3/2008/014.

### Cell culture

Cav-1 KO/WT Mouse Embryonic Fibroblasts (MEFs) were obtained and cultured as described previously [20]. To achieve overexpression of caveolin-1 in adipocytes, fully differentiated 3T3-L1 cells (kind gift of Dr J. Pairault, Paris, FR), were infected with an adenovirus encoding caveolin-1 (cav-1 3T3-L1) or the Green Fluorescent Protein (GFP) as a control. The latter was also used to monitor transfection efficiency. 3T3-L1 cells were maintained and cultured into differentiated adipocytes as described in [20] under 5% CO_2_ atmosphere −37°C during 12 days.

### Isolation of primary adipocytes

Cav-1 KO and WT mature adipocytes were isolated by collagenase digestion of adipose tissue coming from cav-1 KO and WT mice respectively, according to Rodbell [21]. Briefly, the excised fat pads were finely minced into small pieces and digested with collagenase (0.15 U/ml) (Roche, Paris, FR) for 1 h at 37°C under gentle shaking. The cell suspension was then filtered through a 200 µm nylon mesh. Floating cells were collected and gently rinsed twice with Dulbecco's modified Eagle's medium (DMEM) at 37°C and resuspended in the same medium for experiments.

### PCB treatment

Once differentiated into adipocytes, cav-1/control 3T3-L1 and WT/cav-1 KO MEF adipocytes were incubated for 30 min, 90 min, 4 h and 8 h at 37°C with a cocktail of 3 PCB congeners: 2,4,4′-tetrachlorobiphenyl (PCB-28), 2,3′,4,4′,5-pentachlorobiphenyl (PCB-118) and 2,2′,4,4′,5,5′-hexachlorobiphenyl (PCB-153) (Dr Ehrenstorfer, Augsburg, DE). In other experiments, differentiated adipocytes were incubated for 24 hours with the same PCB cocktail and then submitted to a second dose for an additional 8 hours of incubation. In all cultured adipocyte experiments, PCBs were added to the medium as an ethanolic solution at a final concentration of 500 nM for each congener, which is in the range of concentrations found in humans *in vivo* as it lies between the levels quantified in human serum after chronic or acute exposures to PCBs [22], [23]. Control cells received the ethanol vehicle alone that did not represent more than 0.5% (v∶v). Primary adipocytes isolated from cav-1 KO and WT mice were incubated with the same cocktail of PCBs for 2 h at 37°C under gentle shaking. The lactate dehydrogenase assay (Roche), was used to verify that PCB treatments were not cytotoxic (data not shown).

### Isolation of lipid droplets

After incubation of primary adipocytes with PCBs, lipid droplets were isolated as described previously [20]. Briefly, adipocytes were washed twice with PBS and resuspended in 3 ml of disruption buffer (25 mM Tris-HL, 100 mM KCl, 1 mM ethylenediaminetetraacetic acid (EDTA), 5 ml ethyleneglycoltetraacetic acid (EGTA); pH 7.4, further supplemented with a ‘Complete’ protease inhibitor cocktail-one tablet per 25 ml, Roche). Cells were disrupted by nitrogen cavitation at 800 psi for 10 min at 4°C. The lysate was collected and mixed with an equal volume of disruption buffer containing 1.08 M sucrose. It was then sequentially overlaid with 2 ml each of 270 mM sucrose buffer, 135 mM sucrose buffer and Tris/EDTA/EGTA buffer (25 mM Tris-HCl, 1 mM EDTA, 1 mM EGTA; pH 7.4). Following centrifugation at 150 000 g for 60 min, 7 fractions were collected from the top of the gradient.

### Total cell lysates and immunoblotting

Differentiated cav-1/control 3T3-L1 and WT/cav-1 KO MEFs as well as primary adipocytes were lysed as described previously [20]. Cell lysates were then subjected to SDS-PAGE on 12% (w∶v) polyacrylamide gels and transferred onto nitrocellulose membranes (Amersham Biosciences, Munich, DE), blocked for 2 h at room temperature in 5% (w∶v) skimmed milk/Tris buffered saline (TBS, 150 mM NaCl, 50 mM Tris-HCl; pH 7.4) supplemented with 0.1% (v∶v) Tween 20 and probed with primary antibodies against caveolin-1. Nitrocellulose membranes were washed three times in TBS/0.1% (v∶v) Tween-20 for 5 min prior to incubation with secondary peroxidase conjugated antibodies. Protein signals were visualized using enhanced chemiluminescence (Pierce-Perbio biotechnology, Rockford, IL) by exposure to a Kodak X-Omat film.

### Determination of triglyceride concentration

Cells were collected in 300 µl (for MEFs) or 450 µl (for 3T3-L1 adipocytes) of lysis buffer (35 mM sodium dodecyl sulfate, 10 mM EDTA, 60 mM Tris buffer; pH = 7.2). Aliquots of 200 µl were transferred into microtubes followed by the addition of 200 µl of 0.1 M KOH in methanol (Labscan, Gliwice, PL) in a 70°C water bath with gentle shaking for 1 h. Samples were thoroughly vortexed for 10 sec every 20 min. After saponification, the samples were centrifuged at 17 000 g for 10 min. Total released glycerol was then measured by using a sensitive clinical kit according to the manufacturer's recommendations (Free glycerol FS, DiaSys, Holzheim, DE). Glycerol being also present in the backbone of membrane phospholipids, this fraction was quantified by gas chromatography for each cell condition. The quantity of glycerol corresponding to the phospholipids was then substracted from the total amount quantified by the glycerol enzymatic kit after saponification.

### Determination of protein concentration

Cells were collected as described for lipid analyzes. Protein concentration was determined using the Bicinchoninic Acid Protein Assay kit (Sigma-Aldrich, St-Louis, MO), with a bovine serum albumin (BSA) calibration curve.

### Determination of PCB concentrations

Five milliliters of n-hexane (Burdick & Jackson Brand) were added to 4 ml of media or to 300 µl (for MEFs)/450 µl (for 3T3-L1 adipocytes) of cell suspension containing PCBs in an EPA vial (Alltech, Lokeren, BE) for liquid-liquid extraction of the PCBs. After a thorough 5 min shaking, the samples were stored at 4°C until further analysis. All prepared samples (media and cells) were then purified and analyzed as described in Debier et al. [24]. Quantification was performed by comparison with external standards of the analyzed components in a certified calibration mixture (Ultra Scientific and Dr Ehrenstorfer), using a linear calibration curve for each PCB congener (concentration ranging from 1 to 150 pg/µl). The PCB recovery was calculated on the basis of the concentration of the surrogate standard (IUPAC 112, Dr Ehrenstorfer) (50 pg/µl), which was added to the samples at the beginning of the clean-ups.

### Statistical analysis

The statistical analysis of the data was performed by SAS Institute Inc. Software. Comparisons between cell groups were made by two- and one-way ANOVA. Data were considered statistically significant at p-values<0.05.

## Results

### Kinetics of PCB accumulation and intracellular distribution in adipocytes

**Figure 1:**
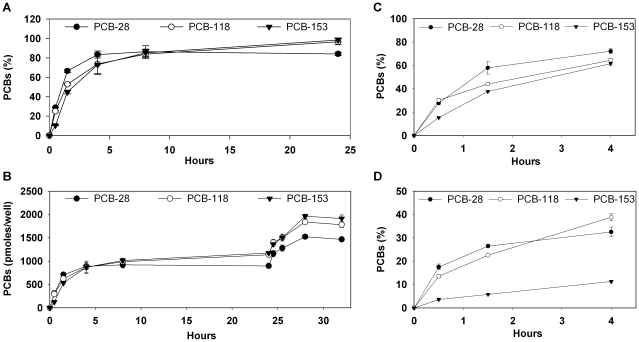
Efficient and differential accumulation patterns of PCBs -28, -118 and -153 in cultured adipocytes. *A*. 3T3-L1 adipocytes were exposed for 24 h to a cocktail of PCBs-28, -118 and -153 (each congener at 500 nM). Cells were collected and quantified for PCB accumulation. Results are expressed as the % of accumulated PCBs in cells as compared to the amounts initially added in the medium. Adipocytes stored nearly the entire dose of PCBs within 24 h. *B*. After a first 24-hour incubation as described in *A*, 3T3-L1 adipocytes were incubated with a second dose of PCBs-28, 118 and 153 added at a final concentration of 500 nM. Cells were collected and quantified for PCB accumulation at each indicated time of incubation. Results are expressed as pmoles of accumulated PCBs in cells. Although already contaminated by the first dose, adipocytes again stored up to 80% of the second amount added in the medium. 3T3-L1 adipocytes (C) and differentiated adipocytes derived from mouse embryonic fibroblasts (MEFs) obtained from WT mice (D) were independently incubated during 4 h with PCBs-28, -118 and -153 added at a final concentration of 500 nM, and quantified for accumulated PCBs. Results are expressed as the % of accumulated PCBs in cells as compared to the amounts initially added in the medium. Each PCB-congener entered the cells with its proper accumulation profile, governed by its own physico-chemical properties.

Differentiated 3T3-L1 adipocytes, an extensively studied cell model for adipocytes, were used to assess time-dependent intracellular accumulation of PCBs in response to an ethanolic cocktail of PCBs-28, -118 and -153, each congener at 500 nM. All congeners were rapidly stored in adipocytes since 45 to 65% of the initially added compounds were already recovered inside the cells after 90 min of incubation. After 24 h, 85% of PCB-28 as well as 98% of PCBs-118 and -153 accumulated in fat cells (Fig. 1A). In all these experiments, media and cells were systematically quantified for residual and accumulated PCBs, respectively, and showed recoveries between 90 and 110%. As a consequence, very little proportion of the added PCB molecules remained in the medium after 24 h, illustrating the very high propensity of adipocytes to take up these compounds and act like sinks to soak them from extracellular space. In order to challenge adipocyte capacity to buffer PCBs, a second dose of the PCB cocktail was added to cells that had been previously exposed for 24 h. Within the following 8 h, up to 80% of each PCB congener present in the second dose had accumulated in the adipocytes (Fig. 1B), indicating an important capacity for PCBs accumulation in cultured fat cells.

Since we observed rapid PCB uptake in 3T3-L1 adipocytes, we evaluated the dynamics of accumulation of each PCB congener over a 4-hour period. PCB-28 accumulated more rapidly in the cells followed by PCB-118 and then PCB-153 (Fig. 1C). MEF derived adipocytes (Fig. 1D) were also tested for PCB accumulation. Interestingly, they accumulated PCBs less extensively than 3T3-L1 cells, since only 30% of PCBs-28 and -118 and 10% of PCB-153 were quantified in cells after 4 h against approximately 70% of each congener in 3T3-L1 adipocytes. Even more obvious differences in accumulation profiles of each congener were observed in MEF derived adipocytes when compared to 3T3-L1s, confirming a marked slower rate of accumulation of PCB-153 in these cells. It is well known that MEFs are less prone to adipocyte differentiation than 3T3-L1 cells, and display lower lipid accretion upon differentiation in culture (triglycerides expressed per unit of total cell proteins were respectively 24±1 ng/µg and 44±3 ng/µg). Considering that MEFs also concentrate less PCBs than 3T3-L1, this might indicate that PCB accumulation by adipocytes is dependent on triglyceride content. Indeed, when the total amounts of accumulated PCBs after 4 hours were expressed per unit of triglycerides in both MEF derived adipocytes and differentiated 3T3-L1 cells, levels were comparable between both cell conditions, respectively 43±6 ng/µg and 38±1 ng/µg (P>0,05), pointing out a possible correlation between the accumulation of PCBs and triglyceride levels in cells. This suggests that these compounds accumulate within the lipid droplet organelle, principally composed of a core of esterified lipid, mainly triglycerides and cholesteryl esters [25] rather than in membranes or other cellular compartments.

**Figure 2:**
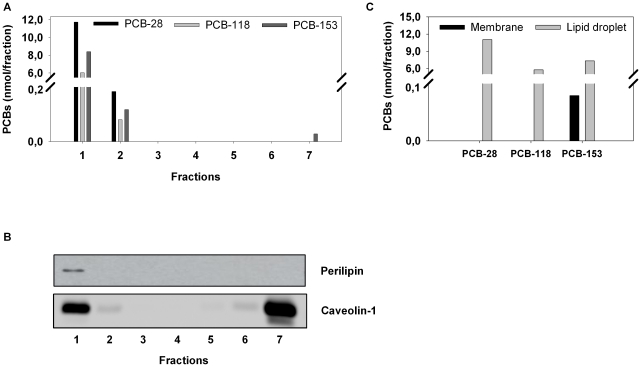
Selective PCB accumulation in lipid droplets of isolated primary adipocytes. Primary adipocytes from cav-1 KO and WT mice were isolated by collagenase treatment and incubated during 2 h with a cocktail of PCB-s 28, -118 and -153 added at a concentration of 30 µM. Lipid droplets were then isolated by sucrose gradient centrifugation. *A*. Quantification of PCBs in all collected fractions (1–7) derived from WT mice. Results are expressed in nmoles of PCBs per fraction and show an almost exclusive association of PCBs with the first fraction corresponding to the lipid droplets. *B*. Western blot analysis of all fractions for caveolin-1 and perilipin indicates the presence of caveolin-1 in both the first (lipid droplets) and last (membranes) fractions. *C.* Association of PCBs with the lipid droplets of primary adipocytes isolated from cav-1 KO mice. Results represent PCB accumulation in the membrane fraction (black bars) and in the lipid droplet fraction (grey bars). Results are expressed in nmoles of PCBs per fraction. PCBs almost exclusively accumulate in the lipid droplets, even in cav-1 KO adipocytes. A small portion of PCB-153 (but not PCB-28 or -118) was found associated to the membranes.

Intracellular PCB distribution was then investigated in mature primary adipocytes that contain large unilocular lipid droplets that can be easily purified by subcellular fractionation on sucrose gradients, as described in Blouin et al [26]. For each congener, up to 98% of total intracellular PCBs were found to be localized in the lipid droplet fractions (fraction 1 and to a much lesser extent fraction 2) (Fig. 2A). No PCBs were found associated with fractions 3 to 6 but 1,5% of PCB-153 was recovered in fraction 7, corresponding to total membranes. Western blot analysis of all fractions (Fig. 2B) confirmed that the lipid droplet marker perilipin was recovered on the top of the gradient. Results show that caveolin-1 was essentially associated with the lipid droplet fraction (fraction 1) and with the membranes (fraction 7).

### Role of caveolin-1 in adipocyte accumulation and lipid droplet targeting of PCBs

**Figure 3:**
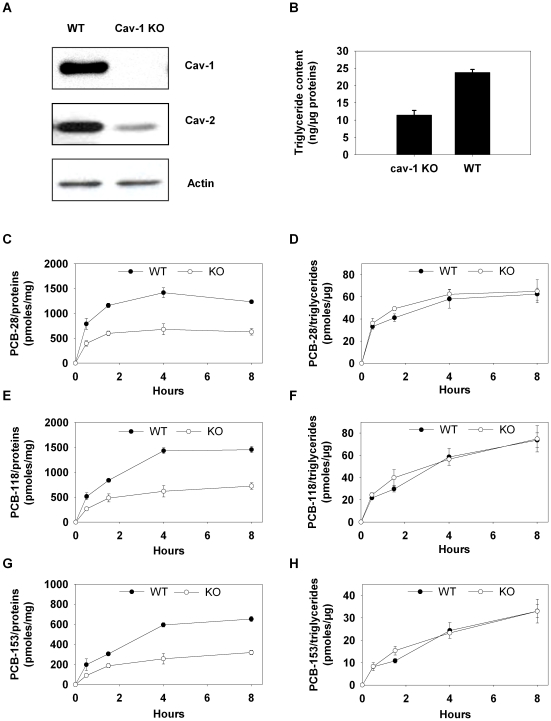
Accumulation of PCBs 28, 118 and 153 in WT and cav-1 KO MEFs. *A.* Western blot analysis for caveolin-1 in MEF differentiated into adipocytes obtained from WT and cav-1 KO mice. Caveolin-1 was not expressed in the knock-out condition. *B.* Images of WT and cav1-KO MEF derived adipocytes illustrating extensive lipid droplet accumulation in the two cell lines. Bar, 10 µm. *C–H.* Differentiated MEFs obtained from WT and cav-1 KO mice were incubated with PCBs-28, -118 and -153 added at a concentration of 500 nM during 8 h, collected and quantified for accumulated PCBs. Accumulated PCBs -28, -118 and -153 are respectively expressed per unit of total cell proteins (C, E, G) or per µg of triglycerides (D, F, H). Although the amounts of accumulated PCBs were significantly different between both cell conditions when expressed per unit of proteins, they became unsignificant when expressed per unit of triglycerides.

We tried to establish whether adipocytes devoid of caveolin expression exhibit altered PCB uptake and/or intracellular distribution. We first treated primary adipocytes from epididymal fat pads derived from cav-1 KO mice for 2 h with cocktail of PCBs and observed that all three congeners almost exclusively distributed in the lipid droplets (Fig. 2C), indicating that cav-1 deficiency does not alter PCB targeting to the lipid storage organelle. In order to examine whether the kinetics of PCB uptake was dependent on caveolin-1, WT and cav-1 KO MEF derived adipocytes were incubated for 8 hours with the PCB cocktail. Western blotting of differentiated WT and cav-1 KO MEFs confirmed the absence of caveolin-1 in the knock-out condition (Fig. 3A) and extensive presence of lipid droplets reflected adipocyte differentiation of the two cell lines (Fig. 3B). Within the 8 hour-incubation period, differentiated WT MEFs accumulated approximately twice more PCBs per unit of cell proteins than their cav-1 KO counterparts (Fig. 3 C, E, G) suggesting a role for caveolin in PCB uptake by adipocytes. However, adipocyte caveolin-1 deficiency has been shown to reduce triglyceride accumulation in adipocytes, causing lipoatrophy in rodents and humans [27], [28]. Accordingly, we observed reduced triglyceride contents in cav-1 deficient adipocytes compared to controls (respectively 11±1 ng/µg protein and 24±1 ng/µg protein) (P<0,0001). Since PCB accumulation occurred within the adipocyte lipid droplet (Fig. 2), we expressed PCB accumulation per unit of triglycerides. This led to comparable PCB uptakes in WT and Cav-1 KO adipocytes (Fig. 3 D, F, H) (p>0.05), and indicates that reduced PCB accumulation in the absence of caveolin might be related to its effect on lipid storage rather than to caveolin-1 presence itself.

**Figure 4:**
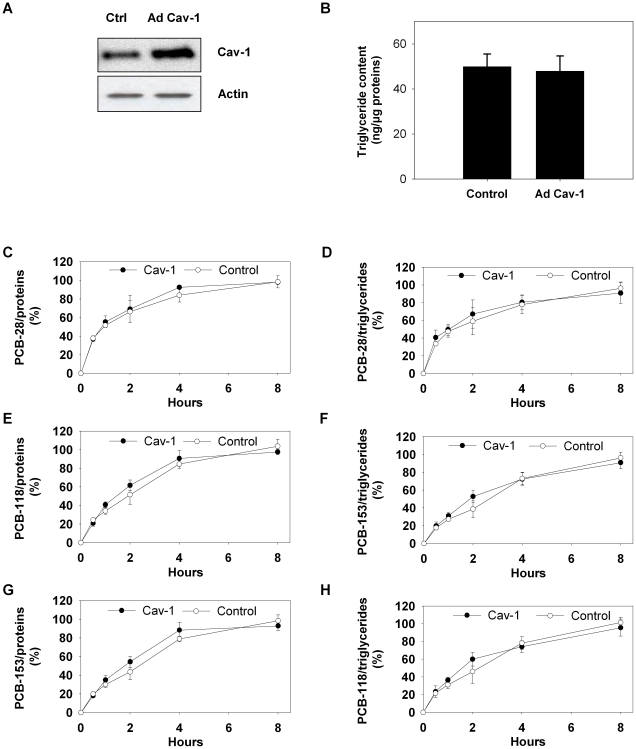
Accumulation of PCBs-28, -118 and -153 in cav-1 overexpressing 3T3-L1 adipocytes and controls. *A*. Western blot analysis for caveolin-1. Differentiated 3T3-L1 adipocytes were infected with an adenovirus encoding for caveolin-1 (Ad Cav-1) or with an adenovirus encoding for the Green Fluorescent Protein (GFP) (Ctrl). Western blot confirms a higher expression of caveolin-1 in the transfected cells as compared to controls. *B.* Cell triglyceride content was quantified in Ad Cav-1 and Ctrl adipocytes. Results are expressed as ng of triglycerides per µg of total cellular proteins and show similar accumulation levels in both cell conditions. *C–H.* Cav-1 or control adipocytes were incubated with PCBs-28, -118 and -153 added at a concentration of 500 nM during 8 h, collected and quantified for accumulated PCBs. Results represent the % of accumulated PCBs expressed per unit of total cell proteins (C, E, G) or per unit of triglycerides (D, F, H). No significant differences were observed in the accumulation levels of PCBs in both cell conditions, when expressed per unit of proteins or per unit of triglycerides.

To assess a direct role of caveolin-1, independent of triglyceride accumulation, we overexpressed cav-1 in 3T3-L1 adipocytes by using an adenovirus. Transient cav-1 overexpression, confirmed by western blotting (Fig. 4A), did not modify triglyceride levels (Fig. 4B) (P>0,05) between controls and cav-1 3T3-L1 adipocytes (50±6 ng/µg protein and 48±7 ng/µg protein, respectively). When expressed per unit of total cell proteins or cell triglycerides, no difference in PCB accumulation was observed upon caveolin overexpression (Fig. 4 C–H) (p>0,05), clearly demonstrating that caveolin had no direct effect on PCB uptake by adipocytes.

## Discussion

Although it is well known that PCBs accumulate in lipid-rich tissues, and thus essentially in the adipose tissue, the mechanisms by which they enter the fat cell and the final destination of the pollutants once inside the cells had not yet been addressed. In the present study, we used several culture models of adipocytes to investigate the mechanisms of PCB accumulation within the adipose tissue. In both 3T3-L1 and MEF derived adipocytes, PCBs were rapidly and efficiently transferred from the culture medium into the cells, reaching concentrations (10 to 70 mg/g of lipids) that are several orders of magnitude higher than in human adipose tissue (20 to 1300 ng/g of lipids) [29], [30]. This substantial difference is quite surprising as the PCB concentrations added to the culture medium were within the range of concentrations found in human serum after chronic or acute exposure. Several factors might explain this phenomenon. Among others, the PCBs administered to 3T3-L1 and MEF adipocytes remained in close contact with the cell monolayer during the entire experimental period, without any circulation or flow in the system. To the contrary, in the *in vivo* situation, PCBs present in the serum are continuously transported through the blood circulation where they are tightly associated with diverse lipoproteins or with plasma albumin [14], [15], [31]. In our experiments, 10% of serum was present in the culture medium of the cells, meaning that the concentrations of lipoproteins or albumin were at least 10 times less important than those found in the blood circulation, probably leading to a lower retention of PCBs in the extracellular compartment. In addition, in the *in vivo* situation, these compounds have to cross the endothelium before being taken up by the adipose tissue that is itself composed of several cell layers. Adipocytes *in vivo* also undergo lipolysis, during periods of negative energy balance, which induces the mobilization of PCBs from the cells. Finally, PCBs may also be taken up by other organs *in vivo*, such as the liver or the skin.

Interestingly, in both culture systems used here, the dynamics of accumulation differed quite importantly between the PCB congeners tested. PCB-28, a *mono-ortho* tri-CB, entered the cells more rapidly than PCB-118, a *mono-ortho* penta-CB, followed later on by PCB-153, a *di-ortho* hexa-CB. These variations likely result from the different molecular structures of the congeners, as the number and the position of the chlorine atoms on the biphenyl structure determine their physical, chemical and biological properties [3], [31]. As far as mechanistic studies are concerned, each PCB congener having its own physico-chemical properties, their behavior towards cells should thus be considered independently from other compounds, even from the same family of pollutants.

An important observation to be drawn from our study is that although the three PCB congeners accumulated in adipocytes with different kinetics, all of them had the lipid droplet as the major final target. This underlines the cell lipid storage organelle as the selective site for PCB intracellular accumulation. One can imagine the lipid droplet as a detoxifying organelle in which organic compounds could be sequestered and kept biologically inactive. Inversely, our observation of massive and selective accumulation of PCBs within lipid droplets could also provide a new basis to understand how these compounds might act to worsen obesity-related metabolic diseases in contaminated humans. Lipid droplets have only recently been identified as highly regulated, complex and dynamic organelles, controlling lipid storage and mobilization [25], [32]. However, the precise cellular interactions between all the regulators involved in the signaling cascades of lipid storage and release remain largely unknown to date. In this line, the possibility that PCBs directly interfere with lipid droplet function, such as fatty acid mobilization from this organelle, should warrant future attention. So far, some *in vivo* and *in vitro* studies highlight a disruption of lipid metabolism by PCBs and other common lipophilic pollutants. Mechanisms involve interference with the lipolysis pathway, impairment of triglyceride synthesis as well as enhancement of adipocyte differentiation and increase in expression levels of diverse enzymes implicated in lipid metabolism or transcription factors regulating energy homeostasis in fat cells [33]–[35]. Taken together, these studies clearly prove that PCBs have an impact on the mechanisms involved in the regulation of cell energy homeostasis and could thus significantly contribute to the development of obesity or obesity-related disorders. The identification, in this work, of lipid droplets as the principal site of PCB concentration in fat cells might thus help to understand the diversity of the biological effects exerted by this family of POPs and certainly deserves more consideration in the future.

It is however important to point out that a very small percentage of PCB-153 (but not PCB-28 nor PCB-118) was localized in the cell membranes. This finding is consistent and in accordance with other studies investigating the potential toxic effects of PCBs through the disruption of cellular membranes in cerebellar granule cell neurons [36], skeletal and cardiac muscle cells [37], rat renal tubular cells [38], mouse thymocytes and lipid bilayer vesicles [39], [40]. All these studies independently reported an increase in cellular membrane fluidity following a treatment with *di-ortho* substituted PCBs (either 2,2′,5,5′-tetrachlorobiphenyl (PCB-52) or PCB-153), but not with other *non-ortho* tested PCB congeners. They show that *di-ortho* congeners dissolve in cell membranes, causing important perturbations to the membrane lipids and proteins and thereby exerting toxic effects on cells. Tan and co-workers (2004) even showed membrane impairments at the mitochondrial and endoplasmic reticulum level in thymocytes and cerebellar granule cells. In our study, as the amounts of PCB-153 associated to the membranes are very small as compared to those found in the lipid droplets, we hypothesize that the pollutants rapidly transferred from the membranes to the lipid droplet triglyceride pools, which are significant in adipocytes as compared to other cell types. However, the fact that a small percentage of PCB-153, but none of the two other congeners, was found associated to the membrane fraction supports the idea that at least part of *di-ortho*-PCB mediated toxicity in the adipose tissue could occur at the membrane level of the fat cell or even at the membrane level of the lipid droplets.

The present study also aimed at identifying specific cell components, abundant in adipocytes, that might be critically involved in the massive accumulation of PCBs in adipose tissue. Starting with caveolin-1 as a candidate, we show here with cell models of genetic loss and gain of function, that caveolin-1 is neither required for the entry of PCBs into cells nor for their accumulation within the lipid droplet organelle. Instead, it appears from our data that the extent of PCB accumulation primarily depends on adipocyte triglyceride content. Indeed, we clearly show that 3T3-L1 adipocytes, which are characterized by higher triglyceride content, accumulated more PCBs at each time of the incubation as compared to MEFs, although the added amounts in the medium were the same. This result is consistent with *in vivo* observations where total body burdens of PCBs are higher in obese than in lean individuals [4], [7]. If dilution capabilities of PCBs are considered, the more triglycerides are present in the fat cell, the more PCBs it can accumulate.

To conclude, our study reveals a massive but differential accumulation of PCBs in cultured adipocytes. The uptake of PCBs was directly linked to the triglyceride content of adipocytes and did not depend on the presence of caveolin-1. The distinct accumulation patterns observed for each congener, as well as the specific association of only PCB-153 to the membranes, points out how important it is to consider a pollutant individually to describe its mechanism of action before describing and understanding the toxicity of mixtures. Eventually, an important conclusion to be drawn from this study is that almost all PCBs were targeted to the lipid droplets in fat cells, an observation suggesting that PCB intra-adipocyte entry and trafficking is tightly linked to lipid metabolism in the adipose tissue. Given the global rise of obesity and its related disorders in humans worldwide, the understanding of the precise impacts of lipophilic pollutants on energy homeostasis in fat cells should be a matter of focus in the future.

## References

[1] Mullerova D, Kopecky J (2007). White adipose tissue: storage and effector site for environmental pollutants.. Physiol Res.

[2] Ulbrich B, Stahlmann R (2004). Developmental toxicity of polychlorinated biphenyls (PCBs): a systematic review of experimental data.. Arch Toxicol.

[3] Carpenter DO (2006). Polychlorinated biphenyls (PCBs): routes of exposure and effects on human health.. Rev Environ Health.

[4] Dirinck E, Jorens PG, Covaci A, Geens T, Roosens L (2011). Obesity and persistent organic pollutants: possible obesogenic effect of organochlorine pesticides and polychlorinated biphenyls.. Obesity.

[5] Hectors T, Vanparys C, van der Ven K, Martens G, Jorens P (2011). Environmental pollutants and type 2 diabetes: a review of mechanisms that can disrupt beta cell function.. Diabetologia.

[6] Lee D-H, Steffes MW, Sjodin A, Jones RS, Needham LL (2011). Low dose organochlorine pesticides and polychlorinated biphenyls predict obesity, dyslipidemia, and insulin resistance among people free of diabetes.. PLoS One.

[7] Kim M-J, Marchand P, Henegar C, Antignac J-P, Alili R (2011). Fate and complex pathogenic effects of dioxins and polychlorinated biphenyls in obese subjects before and after drastic weight loss.. Environ Health Perspect.

[8] Pelletier C, Imbeault P, Tremblay A (2003). Energy balance and pollution by organochlorines and polychlorinated biphenyls.. Obes Rev.

[9] Yu GW, Laseter J, Mylander C (2011). Persistent organic pollutants in serum and several different fat compartments in humans.. J Environ Public Health.

[10] Thorn H, Stenkula KG, Karlsson M, Ortegren U, Nystrom FH (2003). Cell surface orifices of caveolae and localization of caveolin to the necks of caveolae in adipocytes.. Mol Biol Cell.

[11] Couet J, Belanger MM, Roussel E, Drolet M-C (2001). Cell biology of caveolae and caveolin.. Adv Drug Delivery Rev.

[12] Lim EJ, Smart EJ, Toborek M, Hennig B (2007). The role of caveolin-1 in PCB77-induced eNOS phosphorylation in human-derived endothelial cells.. Am J Physiol Heart Circ Physiol.

[13] Lim EJ, Májková Z, Xu S, Bachas L, Arzuaga X (2008). Coplanar polychlorinated biphenyl-induced CYP1A1 is regulated through caveolae signaling in vascular endothelial cells.. Chem-Biol Interact.

[14] Becker MM, Gamble W (1982). Determination of the binding of 2,2′,4,4′,5,5′-hexachlorobiphenyl by low-density lipoprotein and bovine serum albumin.. J Toxicol Environ Health.

[15] Spindler-Vomachka M, Vodicnik MJ, Lech JJ (1984). Transport of 2,4,5,2′,4′,5′-hexachlorobiphenyl by lipoproteins in vivo.. Toxicol Appl Pharmacol.

[16] Uittenbogaard A, Shaul PW, Yuhanna IS, Blair A, Smart EJ (2000). High density lipoprotein prevents oxidized low density lipoprotein-induced inhibition of endothelial nitric-oxide synthase localization and activation in caveolae.. J Biol Chem.

[17] Pilch PF, Liu L (2011). Fat caves: caveolae, lipid trafficking and lipid metabolism in adipocytes.. Trends Endocrinol Metab.

[18] Pol A, Martin S, Fernandez MA, Ingelmo-Torres M, Ferguson C (2005). Cholesterol and fatty acids regulate dynamic caveolin trafficking through the golgi complex and between the cell surface and lipid bodies.. Mol Biol Cell.

[19] Sharma DK, Brown JC, Choudhury A, Peterson TE, Holicky E (2004). Selective stimulation of caveolar endocytosis by glycosphingolipids and cholesterol.. Mol Biol Cell.

[20] Le Lay S, Hajduch E, Lindsay MR, Le Lièpvre X, Thiele C (2006). Cholesterol-induced caveolin targeting to lipid droplets in adipocytes: a role for caveolar endocytosis.. Traffic.

[21] Rodbell M (1964). The Metabolism of Isolated Fat Cells- I: effects of hormones on glucose metabolism and lipolysis.. J Biol Chem.

[22] Wassermann M, Wassermann D, Cucos S, Miller HJ (1979). World PCBs map: storage and effects in man and his bio- logical environment in the 1970s.. Ann NY Acad Sci.

[23] Meeker JD, Maity A, Missmer SA, Williams PL, Mahalingaiah S (2011). Serum concentrations of polychlorinated biphenyls in relation to in vitro fertilization outcomes.. Environ Health Perspect.

[24] Debier C, Pomeroy PP, Dupont C, Joiris C, Comblin V (2003). Quantitative dynamics of PCB transfer from mother to pup during lactation in UK grey seals Halichoerus grypus.. Mar Ecol Prog Ser.

[25] Ducharme NA, Bickel PE (2008). Minireview: Lipid Droplets in Lipogenesis and Lipolysis.. Endocrinology.

[26] Blouin CM, Le Lay S, Eberl A, Köfeler HC, Guerrera IC (2010). Lipid droplet analysis in caveolin-deficient adipocytes: alterations in surface phospholipid composition and maturation defects.. J Lipid Res.

[27] Briand N, Le Lay S, Sessa WC, Ferré P, Dugail I (2011). Distinct roles of endothelial and adipocyte caveolin-1 in macrophage infiltration and adipose tissue metabolic activity.. Diabetes.

[28] Kim CA, Delépine M, Boutet E, El Mourabit H, Le Lay S (2008). Association of a homozygous nonsense caveolin-1 mutation with berardinelli-seip congenital lipodystrophy.. J Clin Endocrinol Metab.

[29] De Saeger S, Sergeant H, Piette M, Bruneel N, Van de Voorde W (2005). Monitoring of polychlorinated biphenyls in Belgian human adipose tissue samples.. Chemosphere.

[30] Wang N, Kong D, Cai D, Shi L, Cao Y (2010). Levels of polychlorinated biphenyls in human adipose tissue samples from southeast China.. Environ Sci Technol.

[31] Matthews HB, Surles JR, Carver JG, Anderson MW (1984). Halogenated biphenyl transport by blood components.. Fundam Appl Toxicol.

[32] Farese RV, Walther TC (2009). Lipid Droplets Finally Get a Little R-E-S-P-E-C-T.. Cell.

[33] Irigaray P, Ogier V, Jacquenet S, Notet V, Sibille P (2006). Benzo[a]pyrene impairs b-adrenergic stimulation of adipose tissue lipolysis and causes weight gain in mice - A novel molecular mechanism of toxicity for a common food pollutant.. FEBS J.

[34] Beranek SR, Becker MM, Kling D, Gamble W (1984). Phospholipid and glyceride biosynthesis in 2, 4, 5, 2′, 4′, 5′-hexachlorobiphenyl-treated human skin fibroblasts.. Environ Res.

[35] Arsenescu V, Arsenescu RI, King V, Swanson H, Cassis LA (2008). Polychlorinated biphenyl-77 induces adipocyte differentiation and proinflammatory adipokines and promotes obesity and atherosclerosis.. Environ Health Perspect.

[36] Kodavanti PR, Shin DS, Tilson HA, Harry GJ (1993). Comparative effects of two polychlorinated biphenyl congeners on calcium homeostasis in rat cerebellar granule cells.. Toxicol Appl Pharmacol.

[37] Wong PW, Pessah IN (1996). Ortho-substituted polychlorinated biphenyls alter calcium regulation by a ryanodine receptor-mediated mechanism: structural specificity toward skeletal- and cardiac-type microsomal calcium release channels.. Mol Pharmacol.

[38] Lopez-Aparicio P, Merino MJ, Sanchez E, Recio MN, Perez-Albarsanz MA (1997). Effect of aroclor 1248 and two pure PCB congeners upon the membrane fluidity of rat renal tubular cell cultures.. Pestic Biochem Physiol.

[39] Yilmaz B, Sandal S, Chen C-H, Carpenter DO (2006). Effects of PCB 52 and PCB 77 on cell viability, [Ca2+]i levels and membrane fluidity in mouse thymocytes.. Toxicology.

[40] Tan Y, Chen CH, David L, Carpenter DO (2004). Ortho-substituted PCBs kill cells by altering membrane structure.. Toxicol Sci.

